# Oxidative Stress in the Tumor Microenvironment and Its Relevance to Cancer Immunotherapy

**DOI:** 10.3390/cancers13050986

**Published:** 2021-02-27

**Authors:** Nada S. Aboelella, Caitlin Brandle, Timothy Kim, Zhi-Chun Ding, Gang Zhou

**Affiliations:** 1 Georgia Cancer Center, Medical College of Georgia, Augusta University, Augusta, GA 30912, USA; naboelella@augusta.edu (N.S.A.); cbrandle@augusta.edu (C.B.); zding@augusta.edu (Z.-C.D.); 2 The Graduate School, Augusta University, Augusta, GA 30912, USA; 3 The Center for Undergraduate Research and Scholarship, Augusta University, Augusta, GA 30912, USA; timkim@augusta.edu; 4 Department of Biochemistry and Molecular Biology, Medical College of Georgia, Augusta University, Augusta, GA 30912, USA; 5 Department of Medicine, Medical College of Georgia, Augusta University, Augusta, GA 30912, USA

**Keywords:** oxidative stress, reactive oxygen species, immunotherapy, tumor microenvironment

## Abstract

**Simple Summary:**

Cancer cells are consistently under oxidative stress, as reflected by elevated basal level of reactive oxygen species (ROS), due to increased metabolism driven by aberrant cell growth. This feature has been exploited to develop therapeutic strategies that control tumor growth by modulating the oxidative stress in tumor cells. This review provides an overview of recent advances in cancer therapies targeting tumor oxidative stress, and highlights the emerging evidence implicating the effectiveness of cancer immunotherapies in intensifying tumor oxidative stress. The promises and challenges of combining ROS-inducing agents with cancer immunotherapy are also discussed.

**Abstract:**

It has been well-established that cancer cells are under constant oxidative stress, as reflected by elevated basal level of reactive oxygen species (ROS), due to increased metabolism driven by aberrant cell growth. Cancer cells can adapt to maintain redox homeostasis through a variety of mechanisms. The prevalent perception about ROS is that they are one of the key drivers promoting tumor initiation, progression, metastasis, and drug resistance. Based on this notion, numerous antioxidants that aim to mitigate tumor oxidative stress have been tested for cancer prevention or treatment, although the effectiveness of this strategy has yet to be established. In recent years, it has been increasingly appreciated that ROS have a complex, multifaceted role in the tumor microenvironment (TME), and that tumor redox can be targeted to amplify oxidative stress inside the tumor to cause tumor destruction. Accumulating evidence indicates that cancer immunotherapies can alter tumor redox to intensify tumor oxidative stress, resulting in ROS-dependent tumor rejection. Herein we review the recent progresses regarding the impact of ROS on cancer cells and various immune cells in the TME, and discuss the emerging ROS-modulating strategies that can be used in combination with cancer immunotherapies to achieve enhanced antitumor effects.

## 1. Introduction

Reactive oxygen species (ROS) are a group of highly reactive oxygen-containing molecules, including free radicals such as hydroxyl (HO^•^), superoxide (O_2_^•^), peroxides (RO^•^) and oxides of nitrogen (NO^•^) and the non-radical hydrogen peroxide (H_2_O_2_). ROS are physiologically generated as a byproduct of cellular respiration and aerobic metabolism, pathologically elevated in diseases like inflammation and cancer, and exogenously formulated after exposure to xenobiotics such as chemotherapy, radiotherapy, or UV. At low to medium levels, ROS can act as cellular signaling messengers, involved in regulating a variety of cellular functions including gene expression, cell proliferation and differentiation, and immunity against diseases. At high levels, ROS cause oxidative damage to DNA, proteins, and lipids, and become detrimental to cells. Due to the multifaceted role of ROS in cell survival and function, the cellular levels of ROS have to be tightly controlled to maintain the redox homeostasis, i.e., the balance between ROS production and scavenging, through multi-layer mechanisms. Oxidative stress occurs when this balance is disrupted in cells. The ontogeny, regulation, and biological function of oxidative stress in cancer biology have been extensively reviewed by others [1,2,3,4]. In this review, we mainly discuss the impact of oxidative stress on the tumor microenvironment (TME), including cancer cells and various immune cells. By focusing on how the interplays between cancer cells and immune cells influence the redox status of both populations, we highlight the therapeutic potential of rational combination of ROS-modulating agents with cancer immunotherapies.

## 2. The Impact of Oxidative Stress on Cancer Cells

It has been well-established that cancer cells are under higher degree of basal level oxidative stress than normal cells, reflected by an increased presence of ROS. Mitochondria are the major cellular source of ROS production. Mitochondria produce ROS during respiration as a natural by-product of electron transport chain (ETC) activity. Incomplete electron transfer and leakage of electrons through ETC complexes I, II, and III results in superoxide production [5]. Membrane-bound NADPH oxidases (NOXs) are another important source of ROS. NOXs are a family of hetero-oligomeric enzymes that catalyze the production of superoxide from O_2_ and NADPH. In most mammals, there are seven NOX isoforms: NOX1, NOX2, NOX3, NOX4, NOX5, dual oxidase (DUOX) 1, and DUOX2 [3,6]. Deregulated ROS generation in cancer cells may occur due to cell-intrinsic events such as oncogene activation, tumor suppressor gene inactivation, increased metabolism, and adaptation to hypoxia (i.e., low oxygen levels), or exogenous insults such as chemotherapy and ionizing radiation [2,3,7,8,9].

### 2.1. ROS in Tumor Initiation, Progression, and Survival

Mildly increased levels of ROS are known to contribute to tumor progression by promoting cell transformation [10], proliferation [11], and survival [12,13,14]. It has been well-documented that growth factor signaling and oncogenic mutations can result in increased ROS production, which is tightly associated with the incidence of various cancers. For example, platelet-derived growth factor (PDGF), epidermal growth factor (EGF), tumor necrosis factor α (TNFα), interleukin-1 (IL-1), transforming growth factor β (TGFβ), etc. can stimulate ROS production and promote tumor progression [15,16,17,18,19]. Oncogenic mutations in RAS have been shown to cause increased generation of superoxide [20,21,22,23]. The oncogene-induced ROS can hyperactivate two important pathways: PI3K/Akt/mTOR and MAPK/ERK signaling cascades [20,24,25]. The PI3K/Akt/mTOR pathway critically regulates cell survival. ROS can activate this pathway by oxidizing and inactivating its negative phosphatase regulators, including phosphatase and tensin homolog (PTEN), protein-tyrosine phosphatase 1B (PTP1B), and protein phosphatase 2 (PP2A), or by the direct oxidation of kinases [26,27]. Many solid tumors, including glioblastoma, melanoma, prostate, and breast cancer, are frequently marked by inactivation of PTEN [28,29,30], suggesting that ROS-induced hyperactivation of the PI3K/Akt survival pathway is critical to the development of these cancers. MAPK signaling pathways are involved in cell growth, differentiation, and survival. Similar to PI3K/Akt/mTOR pathway hyperactivation, ROS induce MAPK/ERK-mediated proliferative signaling through oxidizing and inactivating MAPK phosphatases [31,32]. It is also worth mentioning that ROS can induce nuclear translocation of NF-κB through oxidation and degradation of IκB, the phosphatase inhibitor of NF-κB [33,34,35]. NF-κB is a transcription factor that regulates the genes responsible for inflammation, cell proliferation, differentiation, and survival and is known to promote tumorigenesis, angiogenesis, and metastasis [36,37]. Altogether these studies underscore the correlation between the aberrant cell signaling events and the deregulated ROS generation in cancers [38].

### 2.2. ROS in Tumor Angiogenesis, Metastasis, and Chemoresistance

Increased ROS also facilitate cancer cell angiogenesis [39], metastasis [40,41], and chemoresistance [12]. To meet the increased metabolic needs of proliferating cancer cells, new blood vessels are established to enhance oxygen and nutrient supplies. It is well-known that ROS promote blood vessel formation and angiogenesis [39,42,43]. Tumor hypoxia, a condition in which tumor cells are deprived of oxygen, occurs when tumor growth outpaces blood supply. Hypoxia stimulates the production of mitochondrial ROS (mROS) via the transfer of electrons from ubisemiquinone to molecular oxygen at the Q_o_ site of complex III of the mitochondrial electron transport chain [44,45]. Increased mROS induce and stabilize hypoxia-inducible factor-1 (HIF1a) [44,46,47], a transcription factor that enhances the survival and progression of tumors by upregulating genes regulating tumor angiogenesis, metabolism, metastasis, and chemoresistance [48,49].

Metastasis involves the spread of cancer cells from the primary tumor to the surrounding tissues and to distant organs. Epithelial to mesenchymal transition (EMT) is the process of epithelial cell transition into mesenchymal cell, which is the major cause of tumor metastasis. It has been shown that ROS promote EMT by inducing the expression and activity of certain matrix metalloproteinases (MMPs) that mediate proteolytic degradation of extracellular matrix (ECM) components [50,51]. Cancer stem cells (CSCs) represent a less differentiated but highly tumorigenic subpopulation of cancer cells that contribute to chemoresistance [52]. CSCs are marked by a heightened antioxidant capacity, which allows them to self-renew, differentiate, and importantly, to resist ROS-mediated oxidative damage and cell death induced by radiation or chemotherapy [53,54].

Excessive induction of ROS above a certain threshold can be lethal to the cancer cells [1,2,3]. Cancer cells have an increased antioxidant capacity, mediated by enzymatic and nonenzymatic antioxidants, to adapt to their high oxidative stress status. The main endogenous antioxidant enzymes include superoxide dismutase (SOD), catalase, glutathione peroxidase, glutathione reductase, thioredoxins, peroxiredoxins, etc. The natural nonenzymatic antioxidants include glutathione (GSH), carotenoids, vitamins, etc. One of the vital transcription factors that regulate redox homeostasis in cancer cells is nuclear factor erythroid 2–related factor 2 (NRF2) [55,56]. High levels of ROS prevent the proteasomal degradation of NRF2, thus promoting its nuclear translocation and initiation of the transcription of a multitude of antioxidant genes, including GSH peroxidases (GPXs), and GSH S-transferases (GSTs) [57], glutathione reductase, thioredoxin, thioredoxin reductase, peroxiredoxin and sulfiredoxin. Of note, NRF2 regulates the expression of glutamate-cysteine ligase catalytic (GCLC) and modifier (GCLM) subunits, which combine to form a heterodimer to catalyze the rate-limiting step in GSH biosynthesis. In addition, NRF2 regulates NADPH regeneration enzymes and NAD(P)H:quinone oxidoreductase 1 (NQO1), which inhibits the formation of free radicals by the redox-cycling of quinones [58,59]. NRF2 is therefore considered to be a stress alleviator, supporting cancer cell survival, growth, and escape from the deleterious effects of elevated ROS by maintaining a high but balanced redox status within TME. Moreover, it has been reported that mutations in genes encoding the NRF2 transcription factor and its negative regulator (KEAP1) are frequently detected in cancer [59,60,61]. These mutations may lead to aberrant NRF2 activation, which is associated with poor prognosis and correlates with chemoresistance and tumor recurrence [62,63]. Some recent studies reported that cancer cells can produce neuroglobin (NGB), a monomeric globin, in response to oxidative stress [64]. NGB can act as an oxidative stress sensor and compensatory protein that intersects with the NRF2 pathway to enable tumor cells to resist oxidative stress and acquire chemoresistance [65,66]. Due to its essential role in tumorigenesis, cancer cell proliferation and drug resistance, NRF2 represents a plausible target for anticancer therapy [61,63].

## 3. The Impact of Oxidative Stress on Immune Cells in the TME

The TME is a dynamic environment in which tumor cells reside and interact with the surrounding vasculature, various immune cells, fibroblasts, and ECM. On the one hand, immunosurveillance mediated by T cells and natural killer (NK) cells can detect and attack transformed cells. ROS are important signal mediators involved in the activation of T cells and NK cells. ROS are also used by neutrophils and macrophages to destroy cancer cells. On the other hand, cancer cells possess the ability to induce tumor-promoting immune cells, including regulatory T cells (Tregs), myeloid-derived suppressor cells (MDSCs), tumor-associated macrophages (TAMs), and tumor-associated neutrophils (TANs). The presence of increased ROS and various types of myeloid cells in the TME is characteristic of chronic inflammation, which is intimately intertwined with cancer development and progression [67]. The crosstalk between inflammatory and oxidative stress mediators may form a positive feed-back loop termed “oxinflammation”, shaping the outcome of antitumor immune responses [68]. ROS in the TME, along with other mechanisms, are used by cancer cells and immunosuppressive cells to create immune tolerance to tumors [69,70,71,72,73,74,75,76]. Here, we focus on the impact of ROS on several types of immune cells with relevance to cancer immunotherapy.

### 3.1. The Impact of ROS on T Cells and NK Cells

T cell and NK cell activation leads to an increase in ROS production. It has been well-documented that a mild ROS elevation is required for proper T cell activation and differentiation. ROS act as a secondary messenger participating in the activation of nuclear factor of activated T cells (NFAT) and inhibition of negative regulatory phosphatases to ensure appropriate signaling [77,78]. ROS also contribute to activation induced cell death for T cells to maintain immune homeostasis. Kappler and Marrack reported that activation-induced ROS upregulate Fas expression and downregulate antiapoptotic Bcl2 expression to facilitate T cell apoptosis [79]. The levels of ROS in T and NK cells need to be delicately controlled to avoid the detrimental effects of high levels of ROS. Excessive ROS in T and NK cells can decrease TCRζ- and CD16ζ-chain levels, block NF-kB activation, resulting in deficient IFN-γ, TNF-α, and IL-2 production [80,81,82]. The antioxidative GSH pathway plays a critical role in controlling the redox status in T cells. Mak et al. demonstrated that GSH deficiency in T cells has compromised activation of mammalian target of rapamycin-1 (mTOR) and reduces expression of NFAT and Myc transcription factors, resulting in impaired metabolic integration and reprogramming during inflammatory T cell responses [83]. Tumor-specific T cells treated with the antioxidant N-acetyl cysteine (NAC) during activation significantly reduce DNA damage and cell death, and show improved persistence and antitumor effects upon adoptive transfer into tumor-bearing mice [84,85,86,87]. T cells engineered to co-express a chimeric antigen receptor (CAR) and catalase not only reduce oxidative stress in themselves and exert superior antitumor activity, but also protect bystander NK cells from ROS-mediated repression [88]. Likewise, NK cells primed by IL-15 acquire resistance against oxidative stress through the thioredoxin system, and can aid in protecting other lymphocytes from ROS within the TME [89].

### 3.2. Oxidative Stress and Antigen Presentation

Appropriate levels of ROS are needed for the proper function of antigen-presenting cells. It has been reported that NOX2-mediated phagosomal ROS production in macrophages and dendritic cells (DCs) regulates antigen cross-presentation [90]. Extracellular ROS can also modify the immunogenicity of antigenic peptides, altering T cell priming [91,92]. It has been well-established that induction of oxidative stress in the endoplasmic reticulum (ER) can cause immunogenic death of cancer cells [93,94,95]. Immunogenic cell death (ICD) leads to exhibition and secretion of alarmins, i.e., damage-associated molecular patterns (DAMPs), including adenosine triphosphate (ATP), ER protein calreticulin (CRT) and nuclear heat-shock protein high mobility group box 1 (HMGB1). These DAMPs interact with their receptors (CD91 for CRT, TLR4 for HMGB1, P2RX7 for ATP) on DCs, leading to DC activation, antigen cross-presentation, and ultimately antitumor CD8+ T cell responses. It has been shown that scavenging ROS by antioxidants such as GSH and NAC diminishes ICD [96], whereas strategies that amplify ROS in the ER enhance ICD and augment antitumor immunity [97]. However, there is also evidence that ROS can oxidize the danger signal HMGB1 released from dying cells and thereby neutralize its alarmin activity [98]. A recent study reported that targeted scavenging of extracellular ROS using a tumor ECM targeting nanomaterial can maintain the stimulatory activity of HMGB1 and restore ICD-induced antitumor immunity [99]. These studies suggest that the level and duration of ROS may determine whether or not ICD can occur and lead to effective antitumor immunity.

### 3.3. Oxidative Stress and Immunosuppressor Cells

ROS are not only involved in the induction of Tregs [100], but are also used by Tregs to suppress other immune cells [101,102,103]. Increased numbers of Tregs are often present at tumor sites, indicating that Tregs can persist in this environment despite increased oxidative stress in the TME. Previous studies have shown that Tregs exhibit resistance to oxidative stress, a phenomenon that may be attributed to their increased antioxidative capacity [104,105]. This is further supported by a report showing that GSH-deficiency in Tregs leads to increased serine metabolism, mTOR activation, and proliferation but downregulated FoxP3, resulting in diminished Treg suppressive function in vitro and in vivo [106]. Intriguingly, Tregs sensitive to oxidative stress have recently been described. Maj et al. reported that tumor-infiltrating Tregs tend to undergo apoptosis due to a weak NRF2-associated antioxidant system and resultant vulnerability to oxidative stress in the TME. The apoptotic Tregs convert a large amount of ATP to immunosuppressive adenosine, which antagonizes spontaneous and immune checkpoint blockade (ICB)-induced antitumor T cell immunity [107].

Myeloid cells, including neutrophils, macrophages and MDSCs, are known to produce high amounts of ROS. ROS released by phagocytic cells, mainly neutrophils and macrophages, contribute to tumor killing after chemoimmunotherapy in animal models [108]. However, neutrophils can also use ROS to suppress T cells [109,110]. MDSCs mediate immune suppression via production of ROS and reactive nitrogen species (RNS) [111]. MDSC-derived ROS and RNS reduce T cell responses by inhibiting T cell receptor (TCR) recognition of its ligand, the MHC-peptide complex on target cells [109,112,113]. It is worth mentioning that high levels of ROS in TME promote the maintenance of MDSCs in an immature and immunosuppressive state, while lacking NOX2 [114] or scavenging H_2_O_2_ with catalase [115] promotes immature myeloid cell differentiation into macrophages, resulting in loss of immune suppressive activity of MDSCs. MDSCs are resistant to increased oxidative stress due to an upregulated NRF2-mediated antioxidative system [116], allowing them to exert immunosuppression upon other immune cells through ROS [112,114,117,118,119,120], while protecting themselves from the detrimental effects of ROS.

## 4. Cancer Therapies Targeting Tumor Redox

At different levels, oxidative stress can exert either pro-tumor or anti-tumor effects, presenting as a double-edged sword to cancer cells. Two opposite strategies have been attempted to modulate tumor redox as a way to prevent or treat cancer. One approach is to reduce the tumor-promoting effects of ROS by attenuating oxidative stress using antioxidants. The other approach is to augment cancer cell death by intensifying the levels of ROS in cancer cells. Here we briefly summarize the current status of the two strategies in cancer therapy. More detailed reviews on this subject can be found elsewhere [7,8,9,121,122,123,124,125].

### 4.1. The Use of Antioxidants for Cancer Prevention

Given the role of ROS in promoting tumorigenesis, angiogenesis, and metastasis, various antioxidants have been tested as chemopreventive agents based on the rationale that ROS scavenging can reduce the incidence of cancer and/or delay cancer progression [126]. Gao et al. demonstrated that administration of the antioxidant NAC inhibits tumor incidence in mice by suppressing HIF1a-driven tumor growth [127]. Along the same line, other studies showed that overexpression or targeted delivery of SOD, catalase, or glutathione peroxidase can inhibit tumor growth [128,129,130,131,132]. Although encouraging results were observed in some pre-clinical studies, several large-scale clinical trials with dietary antioxidant supplementation such as vitamin A, vitamin E, and β-carotene failed to demonstrate measurable antitumor benefits [133,134]. Paradoxically, in some cases, antioxidant supplementation has been linked with increased rates of certain cancers [135,136,137]. Possible reasons behind the unexpected failure of the antioxidant approach include inefficient scavenging of tumor-promoting ROS in the relevant cellular compartment such as mitochondria, and/or interference with the antitumor roles of ROS in cancer cells [138,139,140,141,142].

### 4.2. The Use of Pro-Oxidants in Cancer Therapy

Although cancer cells can activate their antioxidant systems to allow them to thrive in the face of increased oxidative stress, they also become more sensitive to further redox disruption. The vulnerability of cancer cells to redox imbalance becomes the Achilles’ heel for cancer. Breaking redox homeostasis in cancer cells can be achieved either by intensifying ROS production or decreasing ROS scavenging through suppressing the antioxidant systems. It is now clear that numerous chemotherapeutic agents exert tumor killing effects through the production of free radicals that cause irreversible cell injury [123,124,143]. Cisplatin, a widely used platinum-based chemotherapy, is known to induce tumor cell apoptosis through generating high levels of cellular superoxide, an effect that can be abolished by the superoxide scavenger Tiron or the antioxidant NAC [144]. 5-fluorouracil (5-FU), an antimetabolite used to treat colon, head, and neck cancers, and other solid tumors, induces tumor cell apoptosis via induction of mitochondrial ROS, and this effect can be blocked by the addition of mitoQ, a mitochondrial-selective antioxidant [145]. Doxorubicin, an anthracycline and topoisomerase inhibitor, induces cancer cell apoptosis as well as cardiotoxicity via direct oxidative DNA damage and indirect induction of H_2_O_2_ [146,147]. Chemotherapeutic agents such as taxanes (paclitaxel and docetaxel) and vinca alkaloids (vincristine and vinblastine) promote the release of cytochrome c from the mitochondria and interfere with the electron transport chain, resulting in the production of superoxide radicals and inducing cell death [148,149,150]. ROS induction also contributes to arsenic trioxide’s potent inhibitory effect on acute promyelocytic leukemia [151]. Buthionine sulfoximine (BSO), an inhibitor of glutamate-cysteine ligase (GCL), the enzyme required for GSH synthesis, exhibits anticancer activity by depleting GSH [152,153]. Some tyrosine kinase inhibitors (TKI) widely used as targeted therapy for cancer, including erlotinib, imatinib, and dasatinib, have been found to induce oxidative stress, which contributes to cancer cell apoptosis, TKI resistance, and cardiac toxicity [154,155]. In addition to these well-established drugs, increasing number of novel compounds, such as beta-phenylethyl isothiocyanate (PEITC) [156], leinamycin [157], and lanperisone (LP) [158], that can act as pro-oxidants or antioxidant inhibitors, have been developed and tested for their anticancer effects. It should be noted that the application of oxidative stress-inducing drugs for cancer treatment also faces many challenges. For example, the use of pro-oxidants may encounter limited tumor-selectivity, dose-limiting toxicity, acquired resistance, and difficulty in effective drug delivery. Besides chemotherapeutic agents, ionizing radiation can also trigger tumor cell apoptosis via ROS induction and the release of mitochondrial cytochrome c [159,160]. Comprehensive summarization of the progress and status of oxidative stress-inducing cancer therapy can be found in other reviews [7,8,9,123,124,125,143].

## 5. The Impact of Cancer Immunotherapies on Oxidative Stress in the TME

In recent years, immune-based therapies, exemplified by ICB therapy and CAR-T cell therapy, have increasingly become a viable treatment option for patients with cancer. Durable and curative outcomes have been observed in a fraction of patients with certain types of cancer after receiving immunotherapies. For example, ICB with aPD1 (nivolumab) and aCTLA4 (ipilimumab) antibodies led to durable responses in ~20% of patients with metastatic melanoma [161], and complete responses were achieved in nearly 80% patients with advanced B-cell acute lymphoblastic leukemia (B-ALL) who received CD19-targeting CAR-T cell therapy [162]. ICB treatment leads to better T cell activation and function by blocking the inhibitory signals transmitted by co-inhibitory molecules such as PD1 and CTLA4. CAR-T cells mediate antitumor effects by specifically recognizing the target molecules on cancer cells and subsequently destroying the cells through cytotoxic granules such as perforin and granzymes, along with inflammatory cytokines such as IFN-γ and TNF-α. Despite their distinct mechanisms of action, emerging evidence from preclinical studies indicates that ICB therapy and CAR-T therapy can both modulate oxidative stress in the TME, and that alteration of tumor oxidative stress contributes to the efficacy of immunotherapy [163,164].

Wang et al. reported that tumor stromal cells, such as fibroblasts, can facilitate tumor chemoresistance by modulating ROS in the TME [165]. GSH and cysteine released by fibroblasts can be used by ovarian cancer cells to diminish nuclear accumulation of platinum, resulting in resistance to platinum-based chemotherapy. The authors showed that tumor-infiltrating CD8 T cells can abolish fibroblast-mediated chemoresistance. Mechanistically, CD8+ T cell-derived IFN-γ reduces extracellular source of GSH and cysteine by upregulating gamma-glutamyl transferases, which break down extracellular GSH, and meanwhile repressing the expression of the cystine and glutamate antiporter system xc^-^ (xCT), which imports extracellular cystine to facilitate GSH synthesis [165]. The same research team further demonstrated that the combination of cyst(e)inase, an engineered enzyme which degrades both cystine and cysteine, and ICB therapy synergistically impairs cystine uptake via xCT in tumor cells, resulting in GSH deficiency, ROS accumulation, lipid peroxidation, and ferroptosis of cancer cells in preclinical models [164]. These studies imply that ROS-driven tumor ferroptosis is an exploitable anti-tumor mechanism, and targeting this pathway in the context of immunotherapy represents a promising therapeutic strategy.

Using mouse tumor models, we reported that adoptive T cell therapy (ACT) can profoundly alter tumor metabolism, resulting in GSH depletion and consequential ROS accumulation in tumor cells [163]. We found that T cell-derived TNFα can synergize with chemotherapy to intensify oxidative stress in cancer cells in a NOX-dependent manner. Reduction of oxidative stress, by preventing TNFα-signaling in tumor cells or scavenging ROS with NAC, antagonizes the therapeutic effects of ACT. Depletion of GSH is one of the mechanisms by which many anticancer drugs elicit ROS-induced tumor cell death [153,156,166]. Our study provides evidence that GSH depletion can be achieved by T cell-based immunotherapy. Unlike most small compound inhibitors, T cell-mediated GSH depletion does not impair the function of the rate-limiting GSH-synthesizing enzyme GCL. However, tumor-specific CD4+ effector T cells can simultaneously disrupt multiple metabolic pathways to cause deficits in several intermediate metabolites involved in GSH synthesis, including homocysteine, cystathionine, and glycine [163]. The overall collapse of the redox-related pathways driven by T cells may block potential compensatory mechanisms, thereby overcoming tumor resistance. These findings imply that the ability of T cells to tilt tumor redox balance toward oxidative destruction is integral to the efficacy of ACT.

The impact of therapeutic antibodies on tumor oxidative stress is examined in a study in which mice bearing implanted lung adenocarcinoma tumors were treated with a cocktail of immunomodulators (anti-PD1, anti-CTLA-4, anti-CD137, and anti-CD19 monoclonal antibodies). Treatment-induced reduction in tumor burden is associated with decreased tumor proliferation but increased oxidative stress, apoptosis, autophagy, and T cell infiltration. The data suggest that treatment with therapeutic antibodies may induce oxidative stress that drives cell cycle arrest and tumor cell death [167].

**Figure 1:**
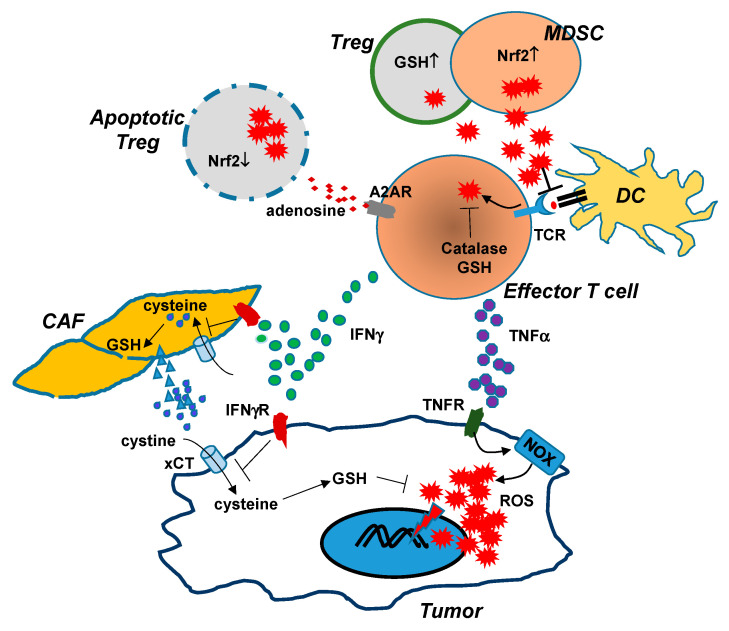
Interactions between cells in the TME lead to changes in redox status. Tumor-reactive effector T cells (CD4+ and CD8+) can induce increased levels of ROS in cancer cells via the actions of IFNγ and TNFα. TNFα signaling in tumor cells activates NADPH oxidases, which lead to increased production of ROS. IFNγ signaling in tumor cells diminishes xCT expression through transcriptional inhibition, reducing tumor uptake of extracellular cystine and subsequent GSH synthesis. IFNγ signaling in cancer-associated fibroblasts (CAF) blocks the release of GSH and cysteine, further depleting the extracellular pool of cystine and cysteine available to cancer cells. The severe redox imbalance in cancer cells, caused by TNFα-driven ROS production and IFNγ-induced GSH deficiency, leads to extensive oxidative damages and eventual tumor cell death. However, effector T cells are also susceptible to oxidative stress in the TME. ROS induced upon TCR engagement are counterbalanced by increased antioxidant systems such as GSH and catalase. T cell dysfunction may occur when effector T cells are exposed to ROS produced by MDSCs and Tregs, which are more resistant to oxidative stress due to their increased antioxidant systems. High levels of extracellular ROS can disrupt antigen-presentation between T cells and DCs, and can affect tumor antigen recognition by T cells. Some apoptosis-prone Tregs can increase the presence of adenosine in the TME, which suppresses the function of effector T cells in an A2AR-dependent manner. Therapeutic interventions should be directed to enhance T cell-induced tumor oxidative stress while enabling T cells to resist the elevated oxidative stress in the TME.

Taken together, accumulating studies start to reveal the cellular and molecular mechanisms by which the dynamic interplay among cancer cells, immune cells, and stromal cells in the TME alters the redox status of each cell population. It is increasingly clear that antitumor T cells possess the ability to induce oxidative stress in tumor cells, and meanwhile they are susceptible to suppression imposed by ROS derived from the surrounding immunosuppressive cells such as Tregs and MDSCs (Figure 1). Therefore, therapeutic strategies should be directed to amplify T cell-induced oxidative stress in cancer cells while relieving effector T cells from the elevated oxidative stress in the TME.

## 6. Emerging ROS-Modulating Agents with the Potential to Enhance the Efficacy of Cancer Immunotherapy

So far, extensive efforts have been focused on developing small molecule compounds or biologics to target certain redox pathways in cancer cells. Although promising results have been observed in some cases, the use of these pro-oxidants for cancer treatment often encounters challenges related to tumor selectivity, toxicity to normal tissues, and development of chemoresistance [7,8,123,124]. We postulate that these issues can be addressed by combining pro-oxidants with immunotherapy in a synergistic manner to achieve durable antitumor effects while minimizing unwanted side-effects. Indeed, the recent findings that increased tumor oxidative stress correlates with the efficacy of ICB and ACT in preclinical models imply that pro-oxidants can be employed to intensify tumor oxidative stress so as to sensitize tumor cells to T cell-based immunotherapy [163,164]. Given that T cells are also sensitive to oxidative stress, it is unlikely any type of pro-oxidant is suitable for combination with immunotherapy. We consider that an immunotherapy-compatible pro-oxidant should meet the following criteria: (1) No obvious toxicity to tumor-reactive T cells at the doses needed to induce oxidative stress in tumor cells; (2) Easy administration to tumor-bearing hosts; (3) Good safety profiles that allow rapid translational studies. Many compounds may satisfy these criteria; here, we only highlight several representative agents which have shown the promise of being able to enhance the efficacy of T cell-based immunotherapy.

### 6.1. High Dose Ascorbate (Vitamin C)

Ascorbate, aka ascorbic acid (AA or vitamin C), at physiological dose functions as an antioxidant. However, mounting evidence indicates that ascorbate used at pharmacological doses (millimolar range) can act as a pro-oxidant that induces extracellular hydrogen peroxide (H_2_O_2_), which can freely diffuse into cells to cause damages in DNA, lipids, and proteins [168,169,170]. Since the uptake of oral ascorbate in humans is tightly controlled by the gut and kidney filtration, pharmacologic concentrations of ascorbate cannot be obtained by oral administration. Intravenous administration of ascorbate bypasses the tight control of the gut and renal excretion, resulting in high levels of ascorbate in plasma. Ascorbate undergoes autoxidation to generate a high flux of extracellular H_2_O_2_. It has been shown that high-dose ascorbate can be tumoricidal in vitro and can inhibit tumor growth in a variety of preclinical models [171,172,173,174,175,176]. Although early randomized clinical trials concluded that oral administration of high-dose ascorbate to patients with advanced cancers does not afford any therapeutic benefits [177,178], this conclusion was later challenged based on the discovery that parenteral (i.v. or i.p.) injection, not oral administration, of ascorbate is required to achieve plasma concentration high enough (20 mM) to damage cancer cells [175,176,179,180,181]. Currently, there are more than 30 completed, recruiting and active clinical trials investigating the usefulness of high-dose ascorbate in cancer treatment, either as monotherapy or in combination with other chemotherapeutic agents (https://clinicaltrials.gov, accessed on 11 February 2021). Completed clinical trials demonstrated that high-dose intravenous ascorbate is well tolerated in cancer patients with normal renal function, and in some cases can alleviate the severity of side-effects caused by chemotherapy [171,175].

The mechanisms underlying the preferential toxicity of ascorbate toward cancer cells over normal cells are not fully understood. One apparent explanation is that the antioxidant systems in cancer cells, which are already overstretched due to increased basal level of ROS, are overwhelmed by the influx of ascorbate-induced H_2_O_2_, while normal cells still have the capacity to mitigate the threat of the H_2_O_2_ burst. Additional mechanisms for ascorbate’s tumoritropic toxicity have also been described [176]. It has been shown that high dose ascorbate can selectively kill human colorectal cancers (CRCs) carrying KRAS or BRAF mutations [173]. This effect is due to increased uptake of the oxidized form of ascorbate, dehydroascorbate (DHA), via the glucose transporter GLUT1. Intracellular DHA is reduced to ascorbate at the expense of intracellular GSH, causing increased oxidative stress in cancer cells. Accumulated ROS inactivate glyceraldehyde 3-phosphate dehydrogenase (GAPDH), an enzyme critically involved in regulating glycolysis, causing an energetic crisis and cell death in highly glycolytic KRAS or BRAF mutant CRC cells but not normal cells. However, another study reported that the selective tumor toxicity by ascorbate is not dependent on DHA uptake. Instead, ascorbate’s toxicity on non-small-cell lung cancer (NSCLC) and glioblastoma (GBM) cells is dependent on the intracellular reactions of H_2_O_2_ and redox-active labile iron [182]. Cancer cells have increased basal levels of O_2_^⋅−^ and H_2_O_2_ [183,184] and increased labile iron [185,186,187]. The increased labile iron in cancer cells leads to increased oxidation of ascorbate to generate more H_2_O_2_ capable of further exacerbating the differences in labile iron in cancer versus normal cells. This self-amplifying labile iron-H_2_O_2_ cycle results in increased Fenton chemistry to generate hydroxyl radicals (•OH) that cause irreversible oxidative damages to cancer cells [182].

It should be noted that ascorbate may also mediate tumoricidal effects through ROS-independent mechanisms. It has been shown that ascorbate is a cofactor for the Ten-Eleven Translocation (TET) enzymes, which mediate DNA demethylation by converting 5-methylcytosine (5 mC) to 5-hydroxymethylcytosine (5 hmC) and other oxidized methylcytosines. Shenoy et al. reported that ascorbate treatment of diffuse large B-cell (DLBCL) and peripheral T-cell (PTCL) lymphomas increases TET activities, which lead to increased demethylation in cancer cells [188]. This epigenetic effect of ascorbate results in reactivation of SMAD1, a tumor suppressor gene, which sensitizes cancer cells to chemotherapy. Along the same line, a subsequent study showed that high dose ascorbate reduces methylation and restores genome-wide 5 hmC levels in clear cell renal cell carcinoma (ccRCC) cell lines via TET activation [189]. Pharmacologic dose ascorbate treatment leads to increased intratumoral 5 hmC and reduced growth of ccRCC in vitro and in vivo. Furthermore, ascorbate treatment has been shown to mimic TET2 activities and suppress human leukemic colony formation and leukemia progression of primary human leukemia PDXs [190]. Of note, the TET-inducing effect of ascorbate is independent of hydrogen peroxide. These data indicate that in addition to its pro-oxidative effect, ascorbate-mediated epigenetic regulation may also contribute to tumor suppression.

**Figure 2:**
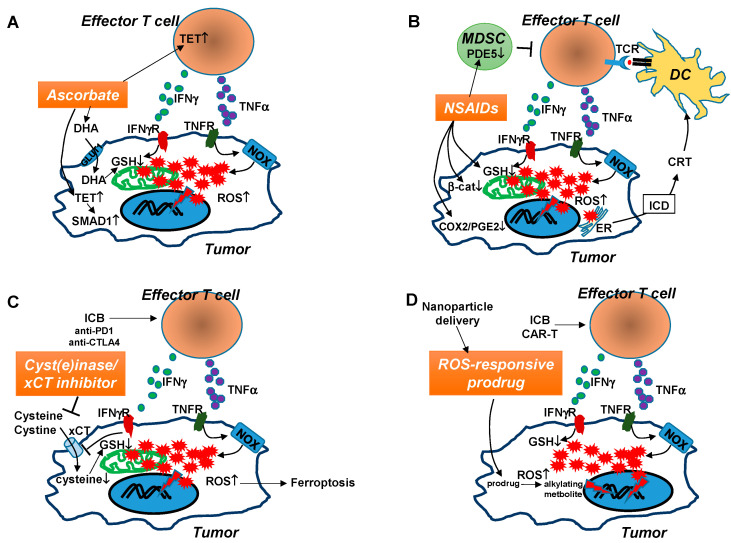
Hypothetical mechanisms by which certain pro-oxidants enhance the efficacy of cancer immunotherapies. Tumor-reactive effector T cells, emerging in the TME after either ICB therapy or adoptive transfer, can mediate tumor killing via well-characterized mechanisms involving cytolytic granules such as perforin and granzymes, and apoptosis-inducing ligands such as FASL and TRAIL. Increasing evidence reveals that inflammatory cytokines produced by effector T cells, including TNFα and IFNγ, can exert antitumor effect by modulating tumor redox. TNFα signaling in cancer cells activates NOX-dependent ROS production, while IFNγ signaling exacerbates GSH deficiency by suppressing the cysteine/glutamate transporter xCT. The combined effects of TNFα and IFNγ lead to substantial ROS accumulation in tumor cells, rendering them vulnerable to further redox disruption which can be incited by a pro-oxidant. A suitable pro-oxidant should preferentially induce oxidative stress in cancer cells without harming antitumor T cells. The potential mechanisms of action of four immunotherapy-compatible pro-oxidants are illustrated. (**A**). Pharmacological dose of ascorbate, in its oxidized form DHA, can be preferentially taken into cancer cells via the glucose transporter (GLUT1). Intracellular DHA is reduced to ascorbate at the expense of GSH. The GSH shortage aggravates ROS accumulation, which damages DNA/protein/lipid and derails cell metabolism, leading to tumor cell death. Meanwhile, ascorbate may induce TET activities in antitumor T cells and tumor cells. TET activation in T cells leads to enhanced function of T cells through epigenetic modifications. TET activation in tumor cells results in demethylation and activation of SMAD1, which increases tumor chemosensitivity. (**B**). NSAIDs can act as pro-oxidants to reduce GSH and thereby increase the levels of ROS in tumor cells. NSAID-induced ER stress may lead to release of calreticulin (CRT), a DAMP molecule characteristic of ICD, which can attract and activate DCs, which in turn elicit antitumor CD8+ T cell responses. In addition, NSAIDs can suppress tumor cell growth by its inhibitory effect on β-catenin, COX2, and PGE2. Some NSAIDs may reduce MDSC activity by inhibiting PDE5 function in MDSCs. (**C**). Cyst(e)inase or xCT inhibitors can reduce the presence or block the uptake of extracellular cystine and cysteine, which tumor cells rely on to synthesize GSH, respectively. Cyst(e)inase can act in concert with ICB-induced antitumor T cells to drive tumor cell ferroptosis. (**D**). ROS-responsive prodrugs can be effectively delivered to tumor loci by nanoparticles. The increased levels of ROS in the TME can activate these prodrugs, which give rise to alkylating metabolites to cause further DNA damage and intensify oxidative stress in tumor cells. These prodrugs may synergize with antitumor T cells because ROS accumulated in tumor cells after immunotherapies such as ICB or CAR-T therapy can effectively activate prodrugs, which in turn further amplify ROS in tumor cells to drive apoptosis.

It is important to note that two recent reports demonstrated that high dose ascorbate synergizes with anti-PD1 ICB therapy in mouse tumor models [191,192]. The two studies showed that administration of high dose ascorbate augments the efficacy of anti-PD1 therapy against several types of cancer in immunocompetent mice. The beneficial effects of ascorbate are associated with enhanced tumor infiltration by CD8+ T cells, granzyme B production by CD8+ T and NK cells, and IL-12 production by antigen-presenting cells. Interestingly, the immunopotentiating effects of high dose ascorbate appear to be independent of its pro-oxidant property. Instead, increased levels of 5 hmC are observed in both cancer and CD8+ T cells, suggesting the involvement of ascorbate-induced TET activities. Importantly, these studies demonstrated that high dose ascorbate does not harm effector T cells, but rather enhances T cell functionality through TET-mediated epigenetic modifications. It is worth noting that in these studies the assumption that the immunopotentiating effects of ascorbate are ROS-independent is based on the observation that provision of antioxidant NAC does not diminish the beneficial effect of ascorbate [192]. Future studies should employ more mechanism-based genetic and/or epigenetic approaches to further determine whether the pro-oxidant effects of ascorbate act in parallel to its epigenetic-modification effects. The possible mechanisms of action of ascorbate and potential combination with immunotherapy are illustrated in Figure 2A.

### 6.2. Non-Steroid Anti-Inflammatory Drugs (NSAIDs)

NSAIDs commonly used for relief of pain, fever, and inflammation act by inhibiting cyclooxygenases (COXs), COX1 and COX2, to suppress prostaglandin synthesis [193]. Some FDA-approved NSAIDs have also been shown to inhibit tumorigenesis in multiple rodent models, and epidemiological studies reported reduced incidence of various cancers in humans, especially colorectal cancer [194,195,196]. Their mechanisms in anticancer activities are not fully understood, but both COX-dependent and -independent pathways play a role. COX2-derived prostaglandin E2 (PGE2) can bind to its receptors on cancer cells and promote tumor cell proliferation, migration, angiogenesis, and chemoresistance [197,198]. Although the anticancer effects of many NSAIDs are attributable to inhibition of the COX2/PGE2 axis, additional mechanisms of action of NSAIDs have been characterized. NSAIDs can inhibit β-catenin transcriptional activity in cancer cells, resulting in reduced tumor growth [199,200]. In addition, suppression of tumor cell growth by some NSAIDs correlates with inhibition of cGMP degrading phosphodiesterase (PDE) activity [201,202]. Furthermore, it has been well-established that NSAIDs can induce oxidative and ER stresses that cause cancer cell apoptosis [203,204,205,206,207,208,209]. A number of commonly used NSAIDs, including sulindac, celecoxib, indomethacin, etc., have been found to induce ROS in various cancer cell lines and can inhibit tumor cell growth independent of COX2 inhibition [210,211,212,213,214]. It has been shown that NSAID treatment destabilizes the redox balance and antioxidant defense mechanisms of the thioredoxin and glutathione systems, resulting in GSH depletion and increased ROS production in tumor cells [215,216,217]. These events lead to a decline in mitochondrial membrane potential, release of cytochrome c, degradation of pro-survival molecules BCL-XL and BCL-2, and activation of the caspase cascade that leads to cancer cell apoptosis [209].

Published studies indicate that the antineoplastic activities of NSAIDs can also provoke antitumor immune responses. Inhibition of PGE2, a potent immunosuppressive factor enriched in the TME, leads to improved antitumor immunity [198,218]. Inhibition of phosphodiesterase 5 (PDE5) activity can abrogate MDSC-mediated immune suppression [219,220]. Suppression of β-catenin can turn immunologic “cold” tumors into “hot” tumors by activating dendritic cells and recruiting T cells into tumors [221,222]. Moreover, NSAID-induced ER stress was reported to correlate with ICD and tumor immunosurveillance [223]. Moreover, tumor antigen-specific T cells stimulated in the presence of a NSAID indomethacin have been shown to acquire stem-like property and can mediate strong antitumor effects upon adoptive transfer in mouse models [224]. These data collectively suggest that certain NSAIDs are compatible with T cell-based immunotherapy. This is supported by the results from an elegant study in which administration of NSAIDs, including celecoxib and aspirin, augments the efficacy of anti-PD1 therapy in multiple mouse tumor models [225]. The beneficial effects of NSAIDs are largely attributed to inhibition of COX2 and PGE2 in this study. It remains to be determined whether the pro-oxidant effect of NSAIDs can enhance immunotherapy for cancers that do not rely heavily on the COX2-PGE2 axis for survival and progression. The development of non-COX inhibitory NSAIDs can also avoid GI and cardiovascular toxicities associated with long-term NSAID administration, reducing potential complications when used in combination with immunotherapy. The possible mechanisms of action of NSAIDs and potential combination with immunotherapy are illustrated in Figure 2B.

### 6.3. xCT Inhibitors and Cyst(e)inase

Cancer cells have a higher demand for GSH to counter balance increased levels of ROS to maintain redox homeostasis for survival and proliferation. Cysteine is the rate-limiting amino acid necessary for GSH biosynthesis. However, under the condition of increased oxidative stress, the production of cysteine in cancer cells is often insufficient to meet the requirements of GSH synthesis, causing cancer cells to rely on uptake of extracellular source of cysteine in its disulfide form cystine via the cystine-glutamate antiporter xCT [226]. Various approaches have been developed to target this pathway as an anticancer strategy [226,227,228,229,230,231]. xCT inhibitors, such as sulfasalazine [232,233,234] and erastin [235,236,237,238,239], can reduce GSH and increase ROS in cancer cells, propagating iron-dependent lipid peroxidation that leads to tumor ferroptosis. Since inhibition of xCT alone may force cancer cells to import cysteine via other amino acid transporters such as alanine/serine/cysteine/threonine transporters (ASCT1 and ASCT2), xCT inhibitors are often used in combination with other chemotherapeutic agents or radiotherapy to overcome drug resistance. An engineered protein cyst(e)inase has been developed to enzymatically degrade both cysteine and cysteine [166]. Administration of cyst(e)inase mediates sustained depletion of the extracellular cysteine and cystine pool in mice and non-human primates. Cyst(e)inase selectively causes cell cycle arrest and death in cancer cells due to depletion of intracellular GSH and ensuing elevated ROS. Cyst(e)inase suppresses the growth of multiple types of cancer in mice, including prostate, breast, leukemia, and pancreatic cancer, yet no apparent toxicities are observed in mice after prolonged treatment [166,240]. These data implicate cyst(e)inase as a safe and effective therapeutic modality for inactivating antioxidant cellular responses in a wide range of malignancies.

Accumulating evidence suggests that xCT inhibitors and cyst(e)inase can be used in combination with immunotherapy to achieve a synergistic antitumor effect. Using genetic approaches, Arensman et al. demonstrated that while xCT is essential for tumor cell growth, it is dispensable for T cell proliferation in vivo and for the generation of primary and memory immune responses to tumors [241]. This study also showed that anti-CTLA4 therapy is more effective in treating xCT-deficient tumors in mouse models, providing proof of concept that administration of xCT inhibitors may augment the antitumor efficacy of ICB. Recent studies from Weiping Zou’s group demonstrated that antitumor CD8+ T cells can drive tumor ferroptosis through IFN-γ-mediated inhibition of xCT, and the combination of cyst(e)inase and ICB therapy synergistically suppresses tumor growth in preclinical models [164,242]. The possible mechanisms of action of cyst(e)inase/xCT inhibitors and potential combination with immunotherapy are illustrated in Figure 2C.

### 6.4. ROS-Responsive Prodrugs

The feature that cancer cells have elevated levels of ROS compared to normal cells has been employed to develop a class of prodrugs that only become cytotoxic in the presence of ROS. Prodrugs that are specifically activated by ROS in tumor cells have the potential to improve tumor selectivity and reduce toxicity to normal tissues. Therapeutic molecules, including chemotherapeutics and anti-PDL1 antibody, can be delivered to and released within tumor cells or TME by ROS-responsive prodrugs or nanoparticles [243,244,245,246,247,248], resulting in significant inhibition of tumor cell growth both in vitro and in vivo. In recent years, several research groups have developed novel prodrugs that act as DNA cross-linking or alkylating agents upon activation by ROS. Leinamycin (LNM) is a potent antitumor antibiotic produced by Streptomyces atroolivaceus S-140. LNM E1 as a prodrug can be oxidatively activated by cellular ROS to generate an intermediate with DNA alkylating activity, exhibiting potent cytotoxicity to prostate cancer cell lines with increased levels of ROS [157]. Peng’s group has developed a series of aromatic nitrogen mustards that are released from prodrugs upon a specific reaction between boronates and H_2_O_2_ in cancer cells [249,250]. These agents show potent DNA cross-linking abilities when coupled with H_2_O_2_, whereas little DNA cross-linking is detected in the absence of H_2_O_2_. These prodrugs can selectively kill leukemia and breast cancer cells, which have inherently high levels of ROS [251]. Interestingly, these prodrugs are not toxic to normal lymphocytes at the doses needed to kill cancer cells [252], suggesting their potential usage in combination with T cell-based immunotherapy. Prodrugs activated via ferrocene-mediated oxidation have also been developed to improve the selectivity of anticancer drugs [253,254,255,256]. These prodrugs show selective toxicity to a variety of cancer cell lines in vitro and in vivo, but remain weakly toxic to nonmalignant cells. Importantly, a recent study demonstrated that one such ROS-responsive prodrug, *N*-(3-(piperidin-1-ylmethyl)benzyl)-4-(ferrocenylcarbamatmethyl)phenyl boronic acid pinacol ester (PipFcB), can sensitize human lymphoma cell lines and primary chronic lymphocytic leukemia cells to CD19CAR-T cells [257]. It is noteworthy that exposure of CAR-T cells to PipFcB does not influence T cell exhaustion, viability, or T cell subpopulations. The T cell-friendly feature of the prodrugs, together with the findings by our group and others that antitumor T cells intensify ROS accumulation in tumor cells, suggest potential synergistic anticancer effects when combining ROS-responsive prodrugs with T cell-based immunotherapies. One possible scenario is that antitumor T cells arising after ICB or CAR-T therapy cause ROS accumulation in cancer cells, which activates prodrugs to release alkylating intermediates, which in turn further amplifies ROS in tumor (Figure 2D). This mutually reinforcing and self-amplifying ROS-inducing loop may lead to specific and complete tumor rejection with minimal toxicities to normal tissues.

## 7. Conclusions and Perspectives

An increasing number of studies indicate that ROS accumulated in tumor cells after immunotherapies are not merely metabolic byproducts but actively contribute to the treatment efficacy. Since these studies were mostly conducted in preclinical models, it remains to be determined in clinical samples whether various forms of cancer immunotherapy, including cancer vaccines, ICB and CAR-T therapy, lead to increased oxidative stress in cancer cells, and whether the levels of tumor oxidative stress correlate with the treatment outcomes. Rational combination of ROS-modulating agents and cancer immunotherapy is emerging as a promising treatment strategy (Figure 2). Given the availability of a multitude of pro-oxidants developed in recent decades, it is possible to identify and utilize a number of novel T cell-compatible agents, such as cyst(e)inase and ROS-responsive prodrugs, to enhance the efficacy of ICB or CAR-T therapy. There are also existing drugs (some are FDA-approved), such as ascorbate and NSAIDs, that can be repurposed as pro-oxidants and used in combination with immunotherapy. Future studies should address the sequence and timing of pro-oxidant administration in relation to immunotherapy, and determine the efficacy and toxicity of the combination therapy in preclinical models with the goal of translating this strategy for the betterment of cancer treatment.
